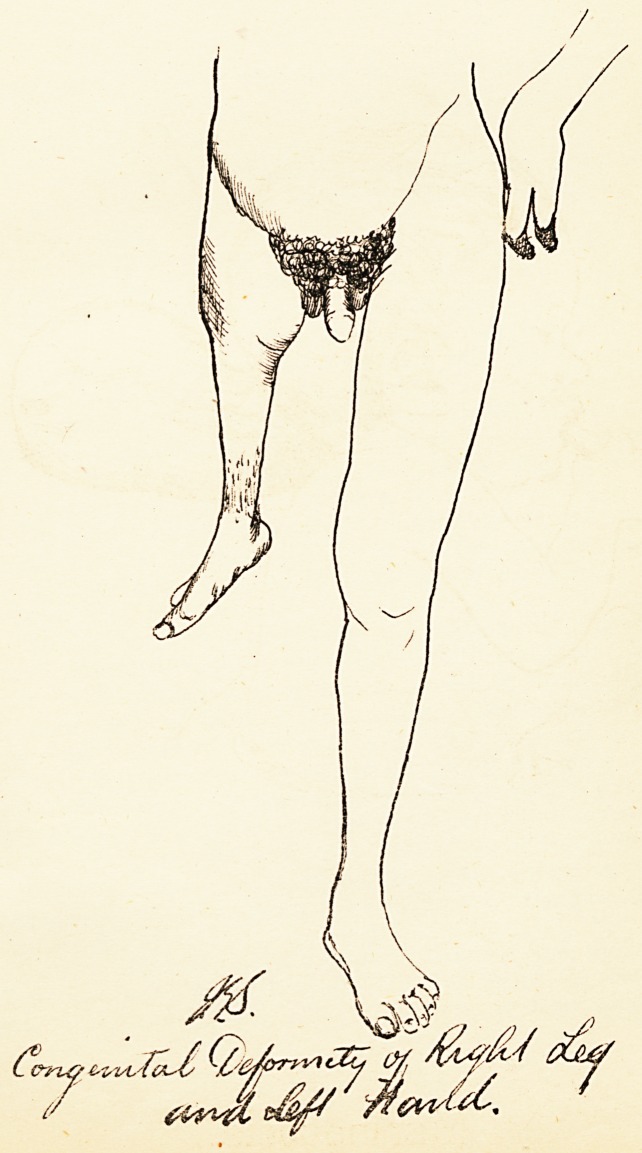# Congenital Deformity of Right Leg and Left Hand

**Published:** 1883-07

**Authors:** 


					CONGENITAL DEFORMITY OF RIGHT LEG
AND LEFT HAND.
The accompanying drawing (PI. XI.), traced from
a photograph, is an example of a rare deformity. The
patient was twenty years of age, and was treated for
typhoid fever in the Bristol Infirmary in 1881. No other
members of his family were deformed.
PlafceXl
/

				

## Figures and Tables

**Figure f1:**